# Supplementary material for: Seminoma and Embryonal Carcinoma Footprints Identified by Analysis of Integrated Genome-Wide Epigenetic and Expression Profiles of Germ Cell Cancer Cell Lines
Source: PLoS One. 2014 Jun 2;9(6):e98330. doi: 10.1371/journal.pone.0098330 (PMC4041891; doi:10.1371/journal.pone.0098330)
Supplement: File S1 — ZIP file containing DMRforPairs output for significant regions. Please start from the html files. (ZIP) [file pone.0098330.s008.zip › figures/8808.pdf]

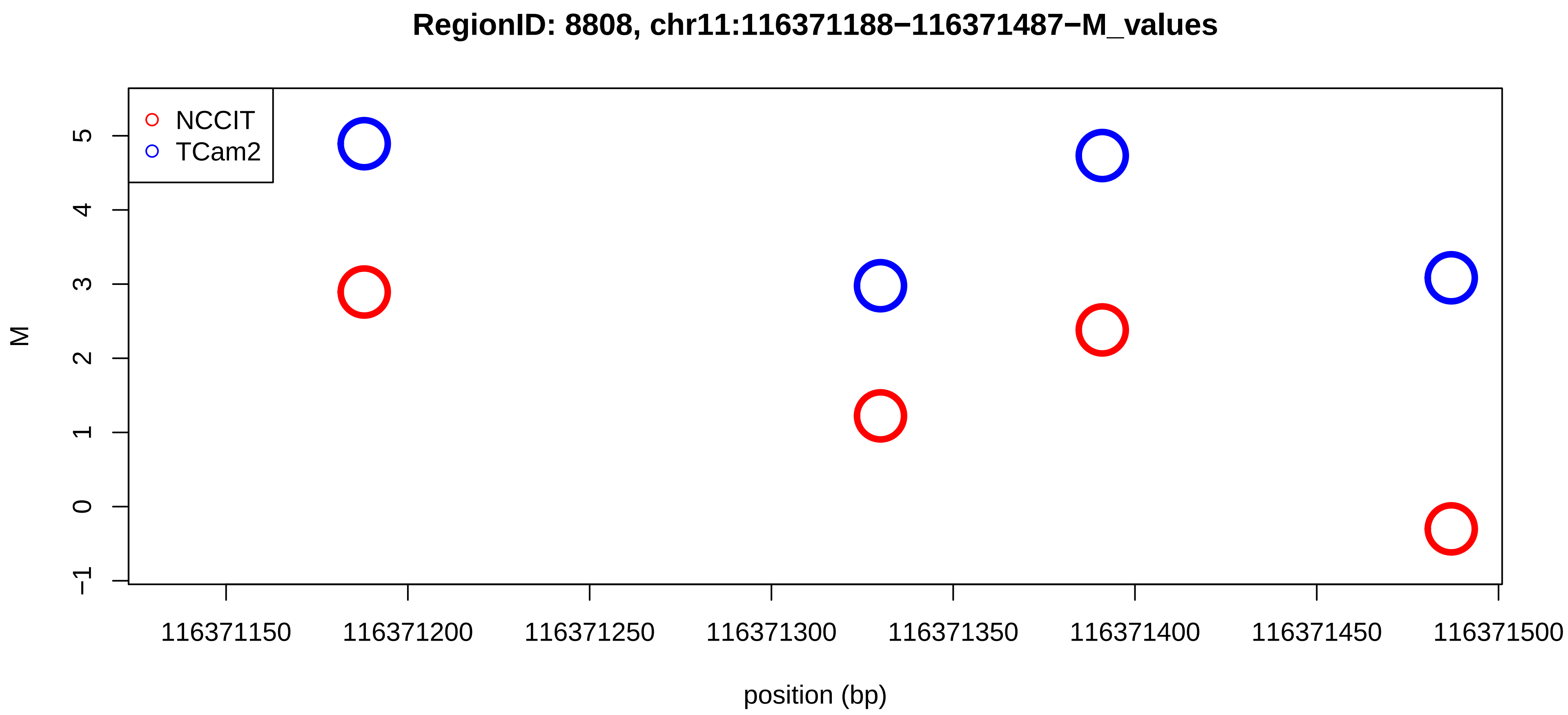
RegionID: 8808, chr11:116371188−116371487−M_values
NCCIT
TCam2
M
position (bp)
116371150
116371200
116371250
116371300
116371350
116371400
116371450
116371500
−1
0
1
2
3
4
5

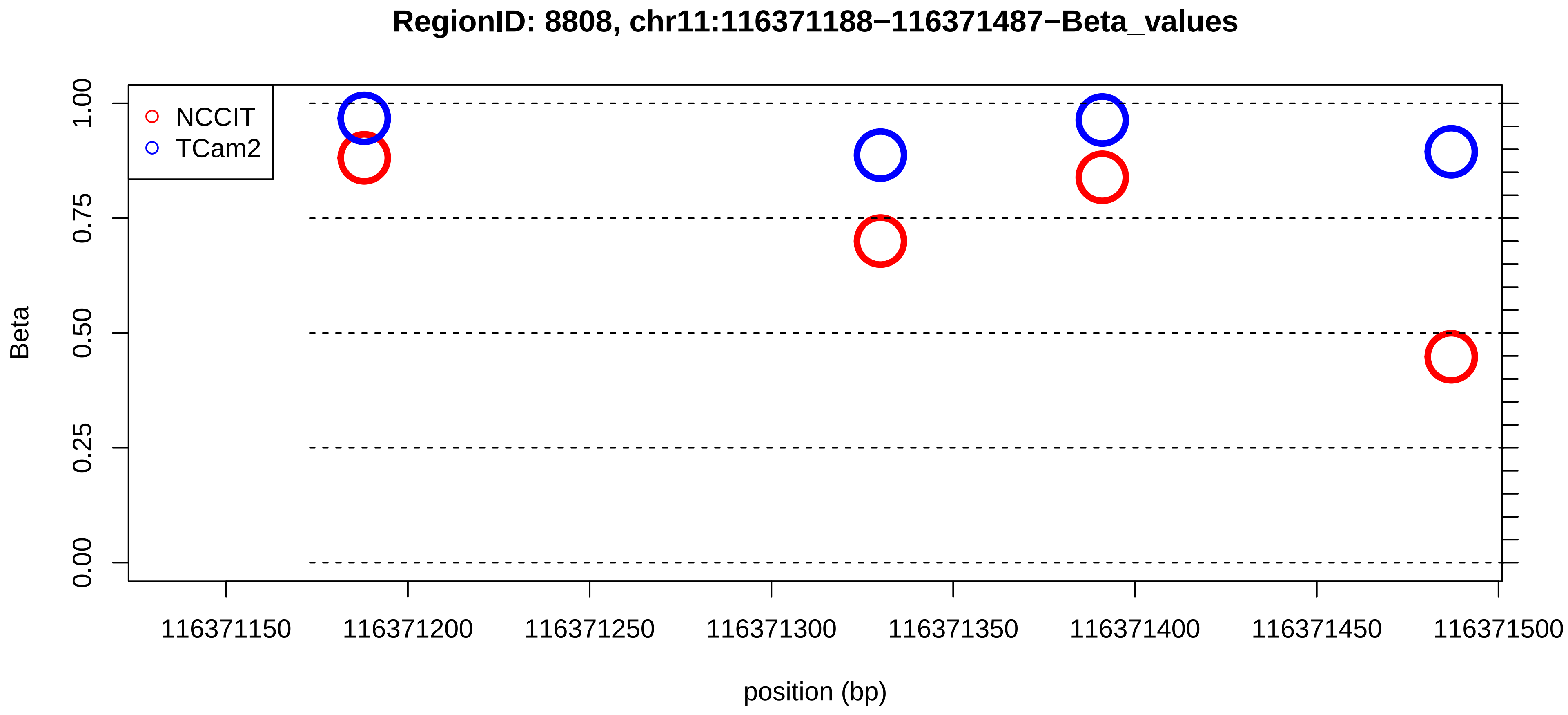
RegionID: 8808, chr11:116371188−116371487−Beta_values
NCCIT
TCam2
Beta
position (bp)
116371150
116371200
116371250
116371300
116371350
116371400
116371450
116371500
0.00
0.25
0.50
0.75
1.00
